# Data from three experiments on auditory attention and distraction in autistic and nonautistic adults

**DOI:** 10.1016/j.dib.2025.112431

**Published:** 2026-01-05

**Authors:** Lejla Alikadic, Jan Philipp Röer

**Affiliations:** Department of Psychology and Psychotherapy, Witten/Herdecke University, Alfred-Herrhausen-Straße 50, 58455 Witten, Germany

**Keywords:** Autism, Selective attention, Auditory distraction, Semantic processing, Working memory, Habituation

## Abstract

In this article, we describe a combined dataset from three experiments on auditory attention and distraction in young adult individuals with Autism Spectrum Disorder (ASD) and nonautisitic individuals. In Experiment 1, we investigated the effects of steady-state, changing-state, and auditory deviant sounds on visual-verbal serial recall with list length adjusted individually to each participant. In Experiment 2, we investigated the effects of low- and high-intensity single-channel, dual-channel, and multi-channel speech on visual-verbal serial recall with a fixed list length of eight to-be-remembered items. Both serial position and cross-trial performance data are available for Experiments 1 and 2. In Experiment 3, we used a selective listening task with a to-be-attended relevant channel and a to-be-ignored irrelevant channel in which the own name and that of a yoked-control partner were embedded. The dataset also contains information on intellectual and language abilities (IST screening scores) and self-report autistic traits (AQ-10). The autistic group was recruited from the same participant pool, so that for many individuals, data from more than one experiment are available that can be compared with each other.

Specifications Table

**Table:** 

Subject	Health Sciences, Medical Sciences & Pharmacology
Specific subject area	Behavioral Sciences: Cognitive in neurodivergent special population, auditory attention and distraction in autism
Type of data	IBM Spss dataset and syntax, Excel dataset, data dictionary
Data collection	Pretests: Intellectual and language abilities were measured using IST screening [1]. Autism severity were measured using the self-reporting short autism questionnaire [2]. Experiment 1: Participants completed a visual-verbal serial recall task on a computer in the laboratory wearing headphones. List lengths were assessed individually. Experiment 2: Participants completed a visual-verbal serial recall task on a computer in the laboratory wearing headphones. Experiment. 3: Participants completed a selective listening task in the laboratory wearing headphones, repeating each word from the relevant message presented to their right ear and ignoring the irrelevant message presented to their left ear.
Data source location	Institution: Witten/Herdecke University Location: Witten, Germany
Data accessibility	Repository name: OSF.io Data identification number: doi:10.17605/OSF.IO/H83T7 Direct URL to data: https://osf.io/h83t7/
Related research article	[3] Alikadic, L., & Röer, J. P. (2024). Contrasting two types of auditory distraction in autistic and neurotypical individuals: A preregistered study. *Research in Autism Spectrum Disorders, 118*(102,493), 102,493. https://doi.org/10.1016/j.rasd.2024.102493

## 1 Value of the Data

• These behavioral data provide valuable insights to the similarities and differences between autistic and nonautistic individuals in ignoring irrelevant auditory information and attending to relevant information. These data are useful for psychologists, physicians, audiologists, and clinical neuroscience researchers interested in exploring basic behavioral research into auditory processing in autism.
• In Experiment 1 and 2, we used a wide range of classic auditory distractor stimuli: (a) Steady-state sequences, (b) changing-state sequences, (c) auditory deviant sequences, (d) single-channel speech, (e) dual-channel speech, and (f) multi-channel speech. A possible avenue of further analysis that we have not looked into is comparing intelligible (single-channel) and unintelligible (dual-channel and multi-channel) speech.
• We were able to replicate several classic findings from the literature including the steady-state effect (increased disruption by steady-state sequences relative to a silent control condition), the changing-state effect (increased disruption by changing-state sequences relative to a silent control or steady-state condition), the auditory deviant effect (increased disruption of auditory deviant sequences relative to a silent control or steady-state condition) [3], and the irrelevant babble effect (increased disruption of single- and dual-channel speech relative to multi-channel speech) [4].
• As far as we know, this is also the first dataset on the cocktail party phenomenon comparing autistic and nonautistic individuals [5]. These specific data could be relevant for researchers interested in hearing-in-noise and speech comprehension.
• Most of the autistic participants took part in all three experiments, allowing for cross-task comparison of working memory (i.e., visual-verbal serial recall under different types of auditory distraction) and selective listening performance and providing a comprehensive view of auditory attention and distraction effects across different paradigms.
• We used individual and fixed list lengths, IST screening scores, and self-reported severity of autism to take the heterogeneity within the autism spectrum into account offering the possibility of correlational analyses of auditory processing, working memory capacity, and verbal intelligence.

## 2 Background

Irrelevant auditory information has the potential to negatively affect cognitive performance. Due to the openness of the auditory system all incoming information is processed to some extent, whether you try to ignore it or not (e.g. [[6], [7], [8]]) Autistic and nonautistic individuals differ in the ability to filter relevant and irrelevant auditory information, which is both of practical and theoretical relevance (e.g. [9,10]; for an overview, see [11]). The overall pattern of results, however, does not paint a clear picture. There is evidence of increased distractibility in autistic individuals compared to nonautistic individuals [12], atypical auditory processing with regard to stimulus complexity, particularly in contexts involving multi-channel speech [13], and mixed findings with regard to the processing of self-relevant information [14].

## 3 Data Description

**Fig. 1:**
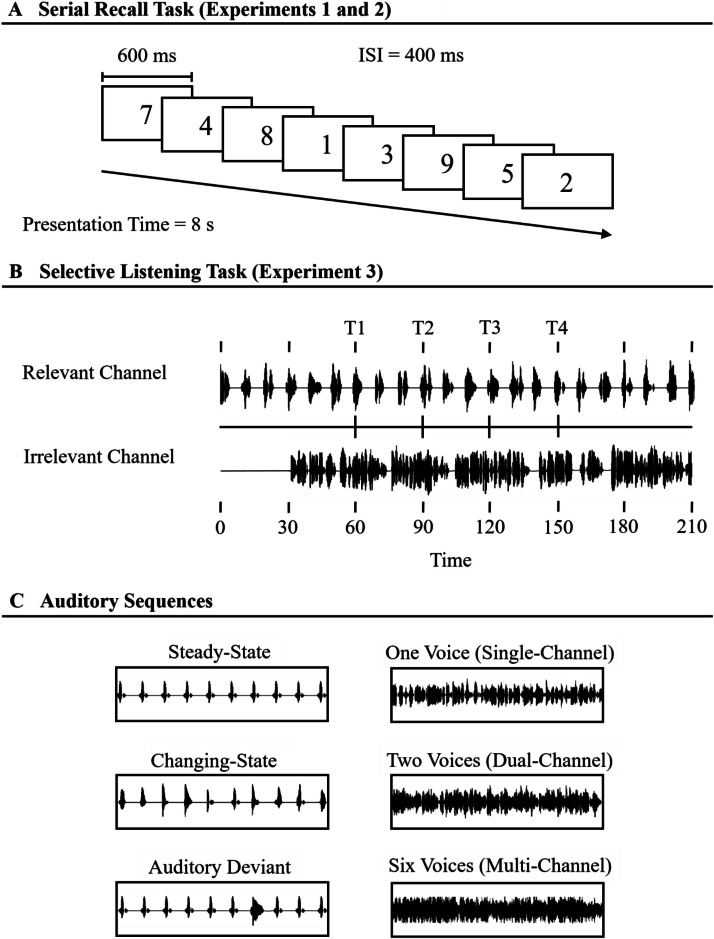
Illustration of the tasks used in Experiments 1 and 2 (panel A), and Experiment 3 (panel B), and sequences presented as auditory distractors in Experiments 1 and 2 (panel C).

Our main goal with the present dataset is to contribute to a more comprehensive understanding of auditory attention and distraction in ASD. We conducted three experiments using established paradigms and a wide range of classic stimuli. To measure the ability to ignore irrelevant auditory information, we used a standard serial recall task. In this task, participants are presented with a list of to-be-remembered digits that are presented one after another on a computer screen while task-irrelevant sound is played over headphones. Participants are informed upfront that all auditory information is irrelevant and that they should ignore it. The serial recall task is the most frequently used paradigm to measure auditory distraction in the lab. We presented six different types of auditory sequences (see Fig. 1 for an overview) allowing us to compare the most important distraction effects in nonautistic and autistic individuals, namely the steady-state effect (e.g. [15]), the changing-state effect (e.g. [7]), the auditory deviant effect (e.g. [6], see also [16]), the irrelevant babble effect (e.g. [17]) and the intensity effect (e.g. [18]). Further, across-trial performance data are available for all auditory conditions, for example to examine habituation effects (e.g. [19]). Serial recall performance was the main dependent variable (i.e., mean recall performance collapsed over all serial positions over all trials in each condition) reflecting the proportion of correct responses.

To measure the ability to attend to relevant auditory information, we used a standard selective listening task. In this task, participants listen to a relevant message presented to one ear and repeat this message word for word and ignore an irrelevant message presented to the other ear. We were specifically interested in whether self-relevant information (i.e., one’s own name) is detected in the irrelevant message, a phenomenon typically referred to as the cocktail party effect ([5], see also [20,21]). The two main dependent variables in Experiment 3 were selective listening performance (i.e., the number of words that were repeated correctly), and shadowing errors on relevant positions (i.e., one word before the name, simultaneously with the name, and one, two, and three words after the name). A systematic and comprehensive investigation of classic auditory attention phenomena contrasting autistic and nonautistic individuals has not yet been conducted (see [3] for a discussion). Therefore we hope that the data we describe here provide valuable insights into the ability to ignore irrelevant auditory information and attend to relevant information in ASD.

All raw and processed data are available at the project website under https://osf.io/h83t7/. There is a combined dataset containing demographic information, IST screening scores, the autism-spectrum quotient, and behavioral data from all experiments and individual datasets for each of the three experiments. Recall performance for Experiment 1 and 2 is available both for each serial position collapsed over trial to calculate serial position curves and for each trial collapsed over serial position to calculate across-trial performance (Combined_Datasets_Serial_Position.sav;

Combined_Datasets_Trial.sav; Dataset_Experiment_1_Serial_Position.sav;

Dataset_Experiment_1_Trial.sav; Dataset_Experiment_2_Serial_Position.sav;

Dataset_Experiment_2_Trial.sav; Dataset_Experiment_3.sav).

We also present syntax files (Syntax_Combined_Datsets.sps) to compute the steady-state effect (contrasting the silent with the steady-state condition), the changing-state effect (contrasting the silent with the changing-state condition, and contrasting the steady-state with the changing-state condition), the auditory deviant effect (contrasting the silent with the auditory deviant condition, and contrasting the steady-state with the auditory deviant condition), the irrelevant babble effect (contrasting the single- and dual-channel with the multi-channel condition), and the intensity effect (contrasting the low-intensity with the high-intensity conditions).

A detailed description of all available data is given in the data dictionary (Data_Dictionary.txt) on the project website. We also present a graphical overview of the file structure (File_Structure.png) for easy navigation.

## 4 Experimental Design, Materials and Methods

### 4.1 Experiment 1

*Participants*. The final sample size of Experiment 1 was 125. The ASD sample consisted of 54 participants (26 female participants, 26 male participants, and 2 participants that answered with “other/not specified”) with a mean age of 28.4 years (*SD* = 6.1). The nonautistic sample consisted of 71 participants (48 female participants, 22 male participants, and 1 participant that answered with “other/not specified”) with a mean age of 22.6 years (*SD* = 3.4). The ASD sample was restricted to the following group of participants: Adult autistic individuals with a maximum age of 39 years and at least one specific diagnosis derived from a clinical professional. Possible diagnoses were: Autism, described by the previously used term 'high functioning', or Asperger's disorder or ASD, defined by the currently used term 'without associated intellectual impairment/without associated language impairment' as described in the diagnostic and statistical manual of mental disorders of the American Psychiatric Association or equivalent in the international statistical classification of diseases and related health problems of the World Health Organization.

*Materials and Procedure.* Prior to the experiment proper, we assessed the severity of autism/autistic traits using a German version of the AQ-10 self-report questionnaire [2], and intellectual and language abilities using IST screening [1]. Individual list lengths of all participants was assessed using a standard serial recall task. Digits were presented at a rate of 1 Hz (800 ms on, 200 ms off) in black font on a white background in the center of the computer screen. The participants' task was to recall the visually presented digits in the correct order as presented, starting with a list length of two. Each block comprised four trials. Prior to each block, participants were informed of the number of digits to be presented. An error was recorded if a digit was not recalled in its correct serial position. When participants made only one error or no error, list length was increased by one digit for the next four trials. This procedure continued until participants made errors in two or more trials within a block. Thus, individual list length was the maximum number of digits that a participant could correctly recall in more than half of the trials.

During the experiment proper, auditory distractor sequences were presented binaurally via closed headphones (beyerdynamic DT 100). Distractors were digitally recorded at a sampling rate of 44.1 kHz with 16-bit encoding. Intensity was measured using a professional hand-held sound level meter that was inserted through the opening of a polystyrene ear. For this purpose, a continuous loop of the distractors was played on the computer on which the experiment was running. In Experiment 1, there were three types of distractor sequences. In the steady-state condition, a single distractor letter was repeated ten times (e.g., B, B, B, B, B, B, B, B, B, B). In the changing-state condition, ten different distractor letters were presented (e.g., J, B, Z, F, L, X, Q, H, S, V). In the auditory deviant condition, the seventh letter of a steady-state condition was replaced by another letter (e.g., B, B, B, B, B, B, Z, B, B, B). Distractors were presented at 65 dB(A). During the silent control condition, no auditory distractors were played.

Twelve trials in each of the four auditory conditions (steady-state, changing-state, auditory deviant, silence) were presented in random order. Immediately after the presentation of the to-be remembered digits, question marks appeared on the screen and participants used the numeric keypad to replace the question marks with the to-be-remembered digits in the order in which they were presented.

### 4.2 Experiment 2

*Participants.* The final sample size of Experiment 2 was 141. The ASD sample consisted of 49 participants (23 female participants, 23 male participants, and 2 participants that answered with “other/not specified”) with a mean age of 29.6 years (*SD* = 6.1). The nonautistic sample consisted of 92 participants (57 female participants, 34 male participants, and 1 participant that answered with “other/not specified”) with a mean age of 23.1 years (*SD* = 3.5).

*Materials and Procedure.* In Experiment 2, the list length of the serial recall task was set at eight items. Auditory distractor sentences in Experiment 2 were sentences extracted from a physiology textbook. There were three types of distractor sequences. In the single-channel condition, one sentence was presented, in the dual-channel condition, two sentences (one female and one male voice) were presented simultaneously, and in the multi-channel condition, six sentences (three female and three male voices) were presented simultaneously. These sequences were played at two intensity levels. In the soft condition, sentences were played at a low intensity of 45 dB(A). In the loud condition, sentences were played at a high intensity of 75 dB(A), which represents an eightfold increase in perceived loudness. Thus, there were six conditions altogether (i.e., soft single-channel, soft dual-channel, soft multi-channel, loud single-channel, loud dual-channel, loud multi-channel). There were 72 trials in total (12 in each condition).

### 4.3 Experiment 3

*Participants.* The final sample size in Experiment 3 was 98. The ASD sample consisted of 48 participants (22 female, 24 male, and 1 participant that answered with “other/not specified”) with a mean age of 29.1 years (*SD* = 6.2). The nonautistic sample consisted of 50 participants (34 female, 15 male, and 1 participant that answered with “other/not specified”) with a mean age of 23.9 years (*SD* = 4.6).

*Materials and Procedure.* A classic selective listening task was used. Participants were instructed to attend to a female voice presented to their right ear (relevant message) via closed headphones (beyerdynamic DT 100) and to repeated each word as soon as they hear it until the sound stops. They were further instructed to ignore the male voice presented to their left ear (irrelevant message). Shadowing errors were recorded by the experimenter, who was sitting at a distance outside the participants' field of vision.

The relevant message consisted of 240 one-, two-, and three-syllable words. The irrelevant message consisted of short sentences, for example proverbs, weather reports, and cooking instructions. Critically, two of the sentences contained the own name of the participants and that of a yoked-control partner, respectively. Name presentation order was counterbalanced across participants. After the selective listening task, participants wrote down everything they could remember from the irrelevant message. They were also asked specifically whether they had noticed their own name or that of the yoked-control partner in the irrelevant message.

## Limitations

The autistic sample was limited to individuals with intellectual and language abilities in the normative range and do not represent the entire autistic spectrum. The nonautistic group consisted of young and healthy psychology students which limits the generalizability of the results. Moreover, groups were not age-matched. On average, the autistic group was five years older than the nonautistic group.

## Ethics Statement

The present research was conducted following the Declaration of Helsinki and received approval from the institutional ethics committee of Witten/Herdecke University (IRB number 189/2021). Prior to their participation, all participants signed written informed consent.

## CRediT Author Statement

**LA:** Conceptualization; Data curation; Formal analysis; Funding acquisition; Investigation; Project administration; Validation; Visualization; Writing – original draft; Writing – review and editing. **JPR:** Conceptualization; Methodology; Resources; Software; Supervision; Visualization; Writing – original draft; Writing – review and editing.
